# EEG datasets for seizure detection and prediction— A review

**DOI:** 10.1002/epi4.12704

**Published:** 2023-02-16

**Authors:** Sheng Wong, Anj Simmons, Jessica Rivera‐Villicana, Scott Barnett, Shobi Sivathamboo, Piero Perucca, Zongyuan Ge, Patrick Kwan, Levin Kuhlmann, Rajesh Vasa, Kon Mouzakis, Terence J. O'Brien

**Affiliations:** ^1^ Applied Artificial Intelligence Institute Deakin University Burwood Victoria Australia; ^2^ Department of Medicine The Royal Melbourne Hospital, The University of Melbourne Parkville Victoria Australia; ^3^ Department of Neurology The Royal Melbourne Hospital Parkville Victoria Australia; ^4^ Department of Neuroscience Central Clinical School, Monash University Melbourne Victoria Australia; ^5^ Department of Neurology Alfred Health Melbourne Victoria Australia; ^6^ Department of Medicine Austin Health, The University of Melbourne Heidelberg Victoria Australia; ^7^ Comprehensive Epilepsy Program Austin Health Heidelberg Victoria Australia; ^8^ Monash eResearch Centre Monash University Clayton Victoria Australia; ^9^ Department of Data Science and AI, Faculty of IT Monash University Clayton Victoria Australia; ^10^ Department of Medicine St Vincent's Hospital, The University of Melbourne Melbourne Victoria Australia

**Keywords:** classification, electroencephalography, machine learning

## Abstract

Electroencephalogram (EEG) datasets from epilepsy patients have been used to develop seizure detection and prediction algorithms using machine learning (ML) techniques with the aim of implementing the learned model in a device. However, the format and structure of publicly available datasets are different from each other, and there is a lack of guidelines on the use of these datasets. This impacts the generatability, generalizability, and reproducibility of the results and findings produced by the studies. In this narrative review, we compiled and compared the different characteristics of the publicly available EEG datasets that are commonly used to develop seizure detection and prediction algorithms. We investigated the advantages and limitations of the characteristics of the EEG datasets. Based on our study, we identified 17 characteristics that make the EEG datasets unique from each other. We also briefly looked into how certain characteristics of the publicly available datasets affect the performance and outcome of a study, as well as the influences it has on the choice of ML techniques and preprocessing steps required to develop seizure detection and prediction algorithms. In conclusion, this study provides a guideline on the choice of publicly available EEG datasets to both clinicians and scientists working to develop a reproducible, generalizable, and effective seizure detection and prediction algorithm.

## 1 INTRODUCTION

The Electroencephalogram (EEG) plays an important role in detecting and localizing seizures, as well as in the diagnosis of epilepsy.^1^, ^2^ The EEG is the most common diagnostic investigation for patients with suspected seizures or epilepsy.^1^ Analyzing EEG using machine learning (ML) techniques has been investigated for seizure prediction and detection,^3^, ^4^, ^5^ with the aim to embed their learned models in monitoring devices that are able to assist epileptologists and other nonspecialist clinicians in settings such as the Epilepsy Monitoring Unit (EMU)^6^ or the Intensive Care Unit (ICU).^7^ These algorithms may also help alert caregivers of impending seizures at home.^8^, ^9^ EEG‐based seizure detection and prediction algorithms proposed by studies over the past 5 years have largely relied on publicly available EEG‐based datasets. However, most of these datasets are not standardized, nor do they come with sufficient information on how and where they should and should not be used. Patient demographics and other relevant clinical data such as epilepsy and seizure classification, device‐specific characteristics, number of channels used, and data collection and segmentation methodology may all affect the performance of prediction and/or detection algorithms, and impact the approach towards their implementation. Some studies report how they consider one or more of the above characteristics of EEG data to inform the design of the data processing and ML pipelines.^10^, ^11^, ^12^ However, this is not common practice, which impacts the reproducibility and generalizability of these findings.

In this paper, we aim to compile and characterize publicly available EEG datasets, and discuss how these characteristics impact the performance and the choice of different methods and ML algorithms. Further, we also investigate and briefly discuss some of the advantages and limitations of the datasets based on the reported characteristics. To the best of our knowledge, there are no reviews that investigate and compare the characteristics of commonly used, publicly available datasets, including those that have been made publicly available in the recent past (<3 years). This paper presents a review of datasets, along with a proposal to streamline datasets into a standardized model.

## 2 BACKGROUND

There are multiple EEG seizure datasets that are publicly available and are used by studies to understand the nature of seizure mechanisms and to develop seizure detection and/or prediction algorithms. However, ML techniques underlying these algorithms are affected by multiple factors such as the duration and sparsity of recorded seizures, sensor placement, and the number of channels included. Seizure events occur in <1% of the total recorded EEG data, which make them highly imbalanced, and often lead to difficulties in prediction or detection tasks using traditional ML approaches. As ML techniques require larger and more balanced datasets, studies often use oversampling and undersampling techniques to overcome this limitation and improve the performance of the ML model.^13^ Having adequately powered datasets with a broad range of seizure types can help reduce the chance of a model misclassifying seizure cases, from out‐of‐sample data (data that are not part of the training process). The type of EEG also plays a vital role in how EEG datasets are used. While scalp EEG is noninvasive, it is less sensitive in detecting electrical synchronization and may contain noise and dropouts when compared to intracranial EEG datasets^1^; although the latter procedure is invasive and expensive, and carries a higher risk of complications.^14^, ^15^ Furthermore, long‐term continuous data, which usually consists of EEG recordings that range from few hours to few days, annotated with seizure start and end times, allows algorithms to consider circadian patterns, and the use of time series models. Given the understanding among research works that seizures are characterized by patient‐specific patterns (as opposed to the clinical approach of grouping patients according to the type of seizures they experience), datasets that are patient‐specific, where the seizure events are labeled with patient identifiers, allow studies to train their algorithm on each patient individually, allowing an algorithm to capture biomarkers and seizure dynamics that are patient‐specific.^16^, ^17^ This allows algorithms to be based on a seizure type or individualized to each patient. Finally, the number of channels present in the dataset might also affect the performance and reproducibility of a model. Including channels that follow the International 10–20 system placement capturing the activity of the entire surface of the scalp allows an algorithm to study and analyze the location and seizure onset. Further, seizure detection or prediction using a dataset that follows a standardized placement and number of EEG electrodes allows other studies to reproduce the result using other datasets.

## 3 DATASETS SEARCH METHODS

**FIGURE 1:**
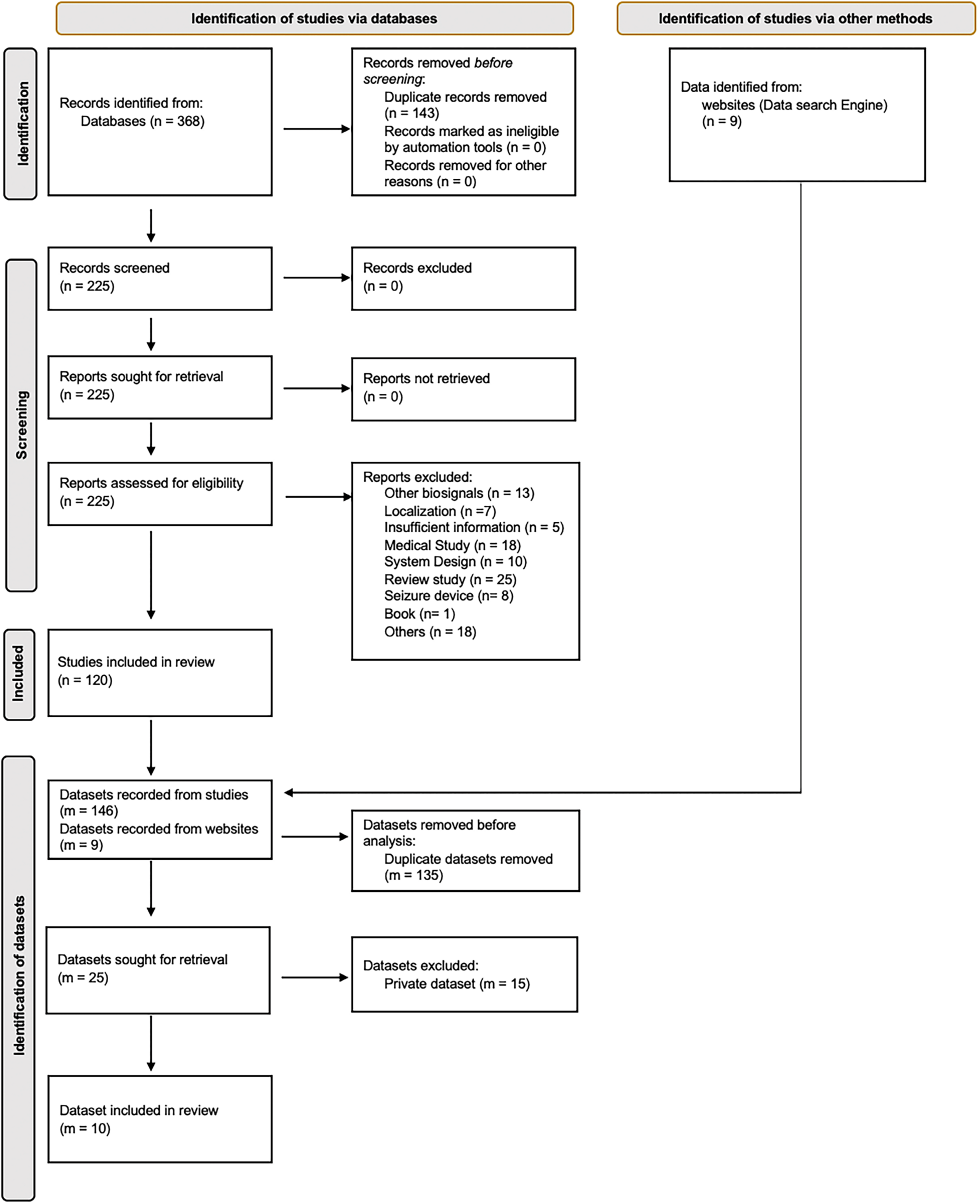
Flow diagram of the dataset search method, identifying publicly available datasets used for seizure detection and prediction, based on the Preferred Reporting Items for Systematic Review and Meta‐Analysis (PRISMA) Guidelines. Abbreviations: *n*—number of studies; *m*—number of datasets.

We adapted the Preferred Reporting Items for Systematic Review and Meta‐Analysis (PRISMA) guidelines to perform a data search as shown in Figure 1. The search of relevant literature that used an EEG seizure dataset was divided into two parts. First, we searched for studies that used EEG signals for seizure detection or prediction on Scopus and Web of Science (WOS), allowing us to include studies from different fields such as medical, information technology, and engineering. Keywords used to search are “seizure prediction” OR “seizure detection.” Due to the high number of relevant studies published over the years, filters were applied to include only studies from the last 5 years with more than 20 citations.

To reduce search errors and information bias, the literature search including studies and data collection was done as follows: The first author labeled the entirety of the results obtained, and the second and third authors labeled 50% of those results each independently. Any disagreements were discussed by the three authors, making the corresponding clarifications of the inclusion and exclusion criteria before the final decision on disagreements was consolidated. Based on the results from the literature search, there were in total of 200 and 168 studies that matched our criteria on Scopus and WOS, respectively. After removing duplicates, there were 225 studies. After applying the exclusion criteria mentioned below, there were 120 studies. We exclude (a) studies with no ML component or insufficient information, (b) studies focused on system design such as the design or architecture of the seizure detection or prediction devices and (c) studies that replicate, validate, or apply existing approaches. Further, we excluded studies that did not examine EEG signals, multimodal signals, or spike detection.

Second, we used the search term “seizure” OR “seizure prediction” OR “seizure detection” OR “epileptic seizure” on Google Dataset Search that was made available in recent years, as well as data repositories such as Kaggle and PhysioNet to find EEG seizure datasets that are publicly available. Review studies on seizure detection and prediction were also reviewed to search for publicly available datasets that we might have potentially missed.^14^, ^18^, ^19^, ^20^ Finally, we aggregated all EEG seizure datasets found from our systematic data search and removed any private or nonaccessible EEG datasets for our analysis.

## 4 RESULTS

**FIGURE 2:**
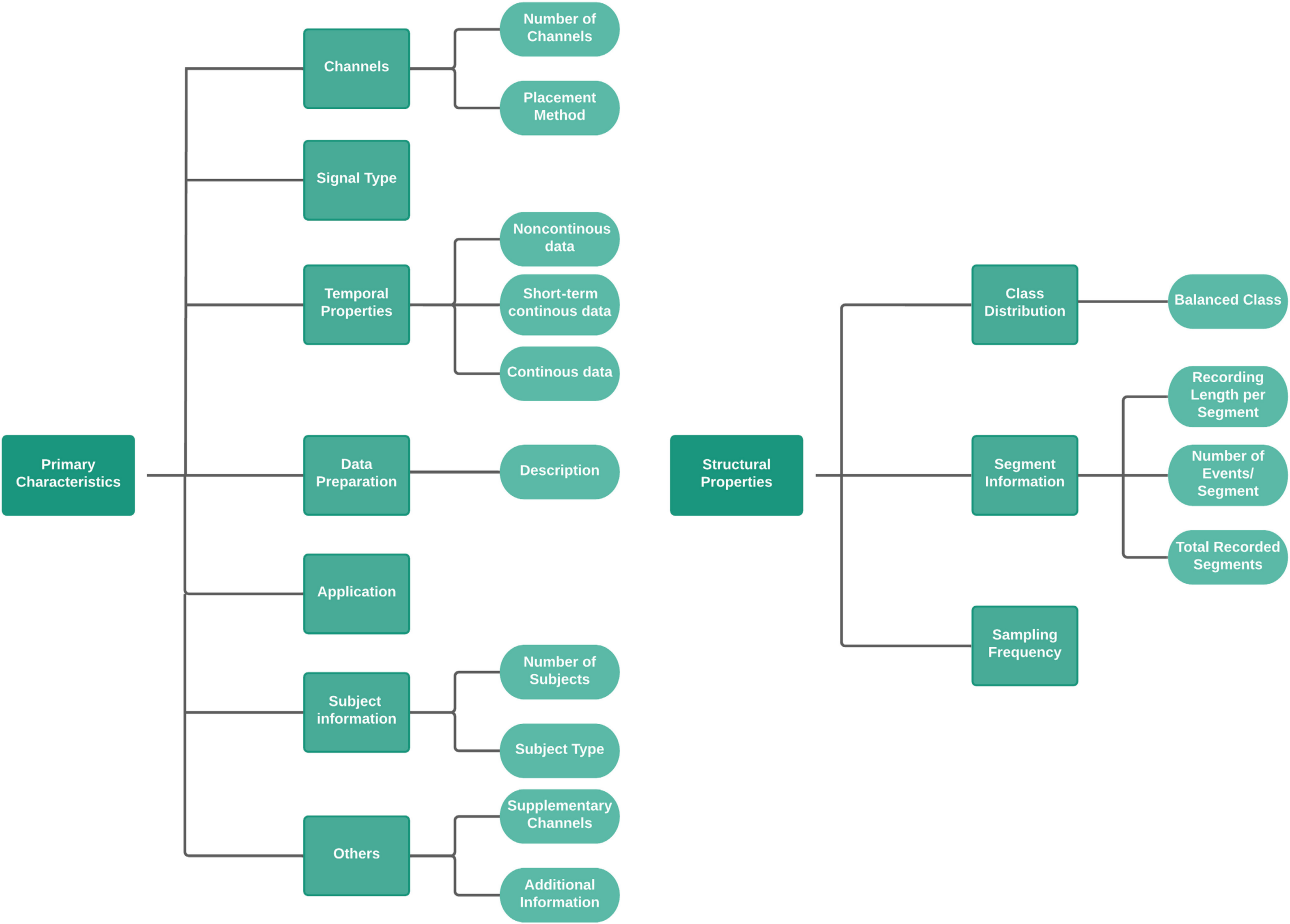
Primary characteristics and structural properties of publicly available datasets. Primary characteristics of a dataset can be subdivided into 7 features (left), while structural properties consist of 3 features (right).

Table 1 shows the publicly available datasets obtained from our data search (Appendix A: Table A1). Datasets obtained from websites through Google Dataset Search, repositories, and review studies include but are not limited to Kaggle dataset,^4^ TUH EEG Seizure corpus (TUSZ),^21^ Siena Scalp EEG and Helsinki University Hospital EEG.^22^, ^23^ However, we will only analyze publicly available EEG datasets, since there is insufficient information provided on private datasets and open access datasets are readily available to researchers. Our analysis of the datasets can be grouped into two key areas as shown in Figure 2, the primary characteristics and the structural properties of the dataset. Both of these key areas provide insights into the differences between various studies using the datasets regardless of the seizure task and will have a direct impact on the methods, as well as ML techniques and the associated limitations. All EEG datasets consist of EEG recordings from patients with epileptic seizures, except for EEG data from the University of Bonn, which also include EEG recordings from 5 healthy subjects with no seizure history.

**TABLE 1:** URL Link to publicly available dataset.

Dataset	URL link
University of Bonn^24^	https://www.ukbonn.de/en/epileptology/workgroups/lehnertz‐workgroup‐neurophysics/downloads/
CHB‐MIT Scalp EEG^53^	https://physionet.org/content/chbmit/1.0.0/
Melbourne‐NeuroVista seizure trial (Neurovista Ictal)^5^	https://melbourne.figshare.com/articles/dataset/Seizure_Data/6939809
Kaggle UPenn and Mayo Clinic's Seizure Detection Challenge^26^	https://www.kaggle.com/competitions/seizure‐detection/data
Neurology and Sleep Centre Hauz Khas^54^	https://www.researchgate.net/publication/308719109_EEG_Epilepsy_Datasets
Kaggle American Epilepsy Society Seizure Prediction Challenge^25^	https://www.kaggle.com/competitions/seizure‐prediction/data
Kaggle Melbourne‐University AES‐MathWorks‐NIH Seizure Prediction Challenge Data^4^	https://www.epilepsyecosystem.org/
TUH EEG Seizure Corpus (TUSZ)^21^	https://isip.piconepress.com/projects/tuh_eeg/html/downloads.shtml
Siena Scalp EEG^22^	https://physionet.org/content/siena‐scalp‐eeg/1.0.0/
Helsinki University Hospital EEG^23^	https://zenodo.org/record/2547147#.Y7eU5uxBwlI

## 5 PRIMARY CHARACTERISTICS

### 5.1 Channels

The diversity of the channels used varied across the datasets as shown in Table 2. Both the University of Bonn and Hauz Khas datasets have only one channel that was cut and selected from continuous EEG recordings from multiple patients to form a single channel that consists of EEG segments from multiple channels. The majority of the datasets consist of 16 to 19 channels. The number of channels in the Siena Scalp EEG dataset is 29, with the exception of one subject who has a 20‐channel EEG recording. Furthermore, datasets like Kaggle UPenn and TUSZ have a varying number of channels among their subjects with huge amounts of variation. The Kaggle UPenn and TUSZ datasets range from 16 to 76 channels and 23 to 31 channels, respectively. It is important to note that all the recordings of canine subjects in the Kaggle UPenn dataset contain 16 channels, while there is a variable number of channels for the patients.

**TABLE 2:** Number of channels, placement method of the channels, and type of signal for the datasets.

Dataset	Number of channels	Placement method	Type of signal
University of Bonn	1	International 10–20 system, Intracranial	Scalp/Intracranial EEG
CHB‐MIT Scalp EEG	18	International 10–20 system/Nomenclature	Scalp EEG
Melbourne‐NeuroVista seizure trial (NeuroVista Ictal)	16	Intracranial	Intracranial EEG
Kaggle UPenn and Mayo Clinic's Seizure Detection Challenge	16–76	Intracranial	Intracranial EEG
Kaggle American Epilepsy Society Seizure Prediction Challenge	16	Intracranial	Intracranial EEG
Neurology and Sleep Centre Hauz Khas	1	International 10–20 System	Scalp EEG
Kaggle Melbourne‐University AES‐MathWorks‐NIH Seizure Prediction Challenge Data	16	Intracranial	Intracranial EEG
TUH EEG Seizure Corpus (TUSZ)	23–31	International 10–20 system/ Nomenclature	Scalp EEG
Helsinki University Hospital EEG	19	International 10–20 system	Scalp EEG
Siena Scalp EEG	20/29	International 10–20 system/Nomenclature	Scalp EEG

Based on Table 2, half of the EEG scalp datasets follow the international 10–20 system electrode placement, while datasets with intracranial EEG use electrodes placed near the epileptogenic zone of the patients, and the remaining datasets contain channels with both the international 10–20 system and the Nomenclature system. The University of Bonn dataset followed the international 10–20 system electrode placement for recording and contains EEG recordings from healthy patients, as well as invasive recordings from patients with epilepsy (in whom electrodes were placed in the epileptogenic zone and hippocampus).^24^

### 5.2 Signal type

Based on Table 2, most of the datasets can be categorized under 2 common types of signal recordings: scalp/surface EEG recordings; or intracranial EEG recordings. Both types of signal recordings are equally distributed among the datasets, with five of the datasets containing scalp EEG and four of the datasets with intracranial EEG. However, the University of Bonn dataset contains a mixture of both scalp and intracranial EEG data where scalp EEG from healthy subjects was taken, while intracranial EEG was taken from subjects with a history of seizures.

### 5.3 Temporal properties

The biggest variation among the datasets surveyed is with regard to temporal properties, as shown in Table 3. Datasets are generally split into multiple segments with the same or different lengths in each segment. The length of each segment is determined by the authors and the limitations of the EEG recording devices. Datasets are split into two categories, continuous and noncontinuous datasets. Continuous datasets are further sub‐categorized as either long‐term (clinical) or short‐term continuous datasets, which can be seen in Table 3. Long‐term EEG continuous datasets consist of more than 24 hours of continuous recording with small time discontinuities in some cases, whereas short‐term EEG datasets contain recordings of less than 24 hours.

**TABLE 3:** Temporal properties of publicly available datasets.

Dataset	Noncontinuous data	Short‐term continuous data	Continuous data
University of Bonn	Yes	No	No
CHB‐MIT Scalp EEG	No	Yes	Yes
Melbourne‐NeuroVista seizure trial (Neurovista Ictal)	N/A	N/A	N/A
Kaggle UPenn and Mayo Clinic's Seizure Detection Challenge	Yes	No	No
Kaggle American Epilepsy Society Seizure Prediction Challenge	Yes	No	No
Neurology and Sleep Centre Hauz Khas	Yes	No	No
Kaggle Melbourne‐University AES‐MathWorks‐NIH Seizure Prediction Challenge Data	Yes	No	No
TUH EEG Seizure Corpus (TUSZ)	No	Yes	No
Helsinki University Hospital EEG	No	Yes	No
Siena Scalp EEG	No	Yes	No

Abbreviation: N/A—not available.

Noncontinuous datasets generally come with random segmentation, where there are no time relationships between each of the neighboring segments. Noncontinuous datasets that are randomly segmented, such as the University of Bonn, Hauz Khas, and Kaggle datasets, are chosen from the unpublished continuous dataset based on multiple criteria depending on the classification tasks.

Short‐term continuous recording datasets such as Helsinki University Hospital EEG contain a routine EEG recording of around an hour, while the Siena Scalp EEG dataset consists of multiple segments of short‐term continuous EEG recording of different recording sessions. The CHB‐MIT Scalp EEG dataset consists of long‐term and short‐term continuous data in segments depending on the patients. However, the dataset has a 10 seconds gap between the continuous segments due to the limitations of the recording device and some longer gaps in certain patients. TUSZ contains multiple pruned short‐term continuous segments from multiple sessions across all patients. Pruning refers to cases where segments that are of interest from the same recording section are cut and joined together. The Neurovista ictal dataset, made available via Epilepsyecosystem.org, does not fall into any category, since it only consists of multiple recordings of seizure events for patients.

### 5.4 Data preparation

Based on Table 4, none of the continuous data, short‐ or long‐term, underwent major preprocessing, except for the different segmentation strategies. Datasets with random segmentation have unique preprocessing steps. Artifacts such as muscle or eye movement were removed from the continuous recording by visual inspection to form the final dataset from the University of Bonn.^24^ In addition to removing artifacts, the EEG segments chosen in the final dataset had to satisfy the criterion of at least weak stationarity.^24^ The only dataset that applied band filtering of between 0.5 and 70 Hz was the Hauz Khas dataset. Each of the segments in TUSZ is pruned to reduce the length of each segment to less than an hour.

**TABLE 4:** A short description of preprocessing steps undergone by the publicly available datasets and its application.

Dataset	Description	Application
University of Bonn	Artifacts removal	Seizure detection
CHB‐MIT Scalp EEG	‐	Seizure detection/Prediction
Melbourne‐NeuroVista seizure trial (NeuroVista Ictal)	Selective segmentation (ictal)	Seizure detection/Prediction
Kaggle UPenn and Mayo Clinic's Seizure Detection Challenge	Selective segmentation:1 h from any seizure for interictal and 4 h from any seizure events for ictal	Seizure detection
Kaggle American Epilepsy Society Seizure Prediction Challenge	Selective segmentation: 5 min before seizure onset and 4 h from any seizure events for preictal, 1 wk minimum from seizure events for interictal	Seizure prediction
Neurology and Sleep Centre Hauz Khas	Frequency filtering between 0.5 and 70 Hz	Seizure detection/Prediction
Kaggle Melbourne‐University AES‐MathWorks‐NIH Seizure Prediction Challenge Data	Selective segmentation: 5 min before seizure onset and 4 h from any seizure events for preictal, 3 h before and 4 h after seizure for interictal.	Seizure prediction
TUH EEG Seizure Corpus (TUSZ)	1 h pruned segment.	Seizure detection/Prediction
Helsinki University Hospital EEG	‐	Seizure detection/Prediction
Siena Scalp EEG	‐	Seizure detection/Prediction

As for the three Kaggle competition datasets, the preprocessing steps and selection criteria for their randomly segmented noncontinuous data are clearer. Interictal segments from both of the datasets for seizure prediction competitions were different, one allows for 1 week minimum and the others select interictal segments hours before and after the seizure events to avoid contamination of preictal and ictal signals, while preictal segments were extracted 5 minutes before the seizure onset to allow sufficient warning time for treatment administration and to account for missed annotations.^4^, ^25^ Similar to the Kaggle seizure prediction competitions, Kaggle UPenn seizure detection dataset only includes ictal segments that are 4 hours away from any other seizure events, and the dataset restricted the randomly segmented interictal data to be 1 hour away from seizures events.^26^

### 5.5 Application

The main task of the study, i.e., whether it is a seizure detection or prediction task, will affect the choice of the dataset. Since the aim of seizure prediction is to forecast impending seizures, EEG recordings that include preictal and interictal data should be used for the study, while the aim of seizure detection is to detect ongoing seizure events, hence, EEG recordings that contain ictal and interictal data should be used. Noncontinuous datasets such as the University of Bonn and Kaggle UPenn datasets only include ictal segments without preictal segments, so they should only be used for seizure detection classification. Similarly, noncontinuous datasets, such as the remaining Kaggle datasets that include only preictal and interictal segments should only be used for seizure prediction classification. However, the noncontinuous Hauz Khas dataset contains preictal, ictal, and interictal events, allowing for its use for both tasks. The Neurovista ictal dataset, which consists of only seizure segments, includes 60 seconds of preictal recordings for each of the seizure segments, which means that it could be used for both seizure prediction and detection, albeit with shorter preictal data. On the other hand, the remaining continuous datasets are more flexible, since the recordings capture preictal, ictal, and interictal events. Hence, these datasets could be used for both tasks. Table 4 also shows the main application that is suitable for the use of the datasets.

### 5.6 Subjects information

Based on Table 5, varying numbers of patient‐specific EEG recordings are present in all of the datasets, except the University of Bonn and Hauz Khas datasets, which are generalized datasets that pool segments of EEG recordings from multiple subjects but do not provide any means to distinguish between patients. Hence, studies that intend to produce algorithms that are patient‐specific, identifying patient‐specific biomarkers, should avoid the University of Bonn and Hauz Khas datasets. The dataset that contains the largest number of subjects is TUSZ with 592, followed by the Helsinki University Hospital and CHB‐MIT datasets with 79 and 23 subjects, respectively. The TUSZ corpus contains the most variation in terms of EEG characteristics in the recordings. While most recordings obtained originated from human subjects, datasets from Kaggle competitions such as Kaggle UPenn and American Epilepsy Society contain EEG recordings of both human and canine subjects.

**TABLE 5:** Number and type of the subjects in the datasets.

Dataset	Number of subjects	Subject type
University of Bonn	10	Human
CHB‐MIT Scalp EEG	23	Human
Melbourne‐NeuroVista seizure trial (NeuroVista Ictal)	12	Human
Kaggle UPenn and Mayo Clinic's Seizure Detection Challenge	12	Human & Canine
Kaggle American Epilepsy Society Seizure Prediction Challenge	7	Human & Canine
Neurology and Sleep Centre Hauz Khas	10	Human
Kaggle Melbourne‐University AES‐MathWorks‐NIH Seizure Prediction Challenge Data	3	Human
TUH EEG Seizure Corpus (TUSZ)	642	Human
Helsinki University Hospital EEG	79	Human
Siena Scalp EEG	14	Human

### 5.7 Others

Additionally, a minority of the datasets included additional information that might be useful for a study. Based on Table 6, TUSZ contains the most complete and diverse patient‐related medical information, including clinical history, medications, EEG characteristics, area of seizure onset, and per‐channel information. Some of the datasets, such as the Siena Scalp dataset, include seizure type and area of seizure onset for each patient. ECG signals were also recorded in the CHB‐MIT Scalp EEG, TUSZ, Helsinki University Hospital, and Siena Scalp EEG datasets. Additional signals such as the vagus nerve signal were recorded in one of the patients for CHB‐MIT Scalp EEG dataset, and photic stimulation was recorded in the TUSZ. Information other than EEG signals is summarized in Table 6.

**TABLE 6:** Other channels that are supplement to the EEG channels and other relevant information that are available in the datasets.

Dataset	Supplementary channels	Additional information
University of Bonn	‐	‐
CHB‐MIT Scalp EEG	ECG, vagus nerve stimulation (partial)	Age, gender
Melbourne‐NeuroVista seizure trial (NeuroVista Ictal)	‐	‐
Kaggle UPenn and Mayo Clinic's Seizure Detection Challenge	‐	‐
Kaggle American Epilepsy Society Seizure Prediction Challenge	‐	‐
Neurology and Sleep Centre Hauz Khas	‐	‐
Kaggle Melbourne‐University AES‐MathWorks‐NIH Seizure Prediction Challenge Data	‐	‐
TUH EEG Seizure Corpus (TUSZ)	ECG and photic stimulation	Medical history, seizure type, EEG characteristics, description, impression, per‐channel information
Helsinki University Hospital EEG	ECG, respiratory effort	‐
Siena Scalp EEG	ECG	Seizure type, origins, age, gender

Abbreviations: ECG—electrocardiography; EEG—electroencephalography.

## 6 STRUCTURAL PROPERTIES

### 6.1 Class distribution

The majority of the datasets consist of two or three types of segments/classes: preictal, ictal, and interictal. The only exception is the University of Bonn dataset, which contains five types of segments; the first two sets contain EEG data from the surface of healthy subjects both with eyes open and closed. The remaining types of segments from epilepsy patients contain seizure‐free recordings from the epileptogenic zone and hippocampal zone. Since seizures do not occur often enough and are rare during the limited time of the EEG recording period where each seizure only occurs for several minutes, not all seizures are captured. Hence, the problem of class imbalance also exists in most of the datasets where the minority classes are preictal and ictal segments. Based on Table 7, the exceptions are the University of Bonn, where all five segments are balanced, and the Hauz Khas dataset where the number of preictal, ictal, and nonictal segments are balanced. Interestingly, both of the datasets contain EEG segments that are noncontinuous and are chosen from a full recording in order to achieve a more balanced dataset, which does not represent true seizure dynamism. The Neurovista ictal dataset, which consists of seizure events for all patients, includes 60 seconds of preictal data and 10 seconds of seizure offset data and is unique in this case, since segments contain a majority of seizure recordings.

**TABLE 7:** The distribution of preictal, ictal, and interictal class in the datasets.

Dataset	Balanced class
University of Bonn	Yes
CHB‐MIT Scalp EEG	No
Melbourne‐NeuroVista seizure trial (NeuroVista Ictal)	No
Kaggle UPenn and Mayo Clinic's Seizure Detection Challenge	No
Kaggle American Epilepsy Society Seizure Prediction Challenge	No
Neurology and Sleep Centre Hauz Khas	Yes
Kaggle Melbourne‐University AES‐MathWorks‐NIH Seizure Prediction Challenge Data	No
TUH EEG Seizure Corpus (TUSZ)	No
Helsinki University Hospital EEG	No
Siena Scalp EEG	No

### 6.2 Segment information

Regarding the temporal properties of EEG datasets, the length of segments varies across all datasets for both continuous and noncontinuous datasets as shown in Table 8. Within the same dataset itself, the length of each segment is different where each seizure event can be divided into multiple ictal or preictal segments. This can be seen in Table 8, where the Helsinki University Hospital EEG dataset has a median epoch length of 74 minutes, and the Neurovista ictal dataset has different seizure segment lengths, with an average of 107 seconds. Further, TUSZ has less than 1 hour of pruned segments and various epoch lengths (minutes to hours) across patients in the Siena Scalp EEG dataset. The other exception is the CHB‐MIT dataset with 1‐hour length except for six patients, and occasional shorter segment lengths. The other datasets had a majority of segments with identical length, with the shortest being 1‐second segments in Kaggle UPenn dataset and the longest segments of 10 minutes in both of the Kaggle seizure prediction datasets.

**TABLE 8:** Segment information including temporal properties, number, and total recorded segments of the datasets and its sampling frequency.

Dataset	Recording length per segment	Number of events/segments	Total recorded segments	Sampling frequency (Hz)
University of Bonn	23.6 s	500 segments	500	173.86
CHB‐MIT Scalp EEG	Typically, 1 h	198 events	664	256
Melbourne‐NeuroVista seizure trial (NeuroVista Ictal)	Average of 107 s	2979 segments	2979	400
Kaggle UPenn and Mayo Clinic's Seizure Detection Challenge	1 s	48 events	25 922	400–5000
Kaggle American Epilepsy Society Seizure Prediction Challenge	10 min	111 events	4067	400–5000
Neurology and Sleep Centre Hauz Khas	5.12 s	100 segments	150	200
Kaggle Melbourne‐University AES‐MathWorks‐NIH Seizure Prediction Challenge Data	10 min	633 segments	5047	400
TUH EEG Seizure Corpus (TUSZ)	1 h	3050 events	5610	Minimum of 250
Helsinki University Hospital EEG	Median of 74 m	460 events	79	256
Siena Scalp EEG	varied	47 events	41	512

Abbreviation: Hz—Hertz.

The University of Bonn and Hauz Khas datasets did not have information regarding the number of seizures captured, so ictal segments were used in this case. The University of Bonn consists of 100 ictal segments, while Hauz Khas has 50 ictal segments and 50 preictal segments. The Neurovista ictal dataset, which only includes ictal events, has a total of 2979 segments. TUSZ has the highest number of seizure events at 3050, followed by The Kaggle competition dataset from Melbourne University and Helsinki University Hospital EEG at 633 and 460 seizure events, respectively. The Kaggle UPenn and Siena Scalp EEG datasets have the lowest seizure events recorded at 48 and 47, respectively.

The Neurovista ictal dataset, which contains only ictal data, has the same number of segments as ictal events at 2979. Kaggle UPenn's dataset has the highest total recorded segments at 25 922 from 12 subjects with the lowest recording length at 1 second. This is followed by TUSZ with 5610 recorded segments with pruned 1‐hour recordings from 642 patients and Kaggle Melbourne University competition at 5047 ten‐minute segments from 3 patients. The Siena Scalp EEG dataset contains the lowest number of total recorded segments at 41 from 14 patients followed by Helsinki University Hospital EEG dataset, with a one‐to‐one mapping between recorded segments and subjects, i.e., 79 recorded segments from 79 neonates.

Based on Table 8, the number of seizure events is largely affected by the total size of the dataset (Total recorded segments x recordings length per segment). Datasets such as the Kaggle competition from Melbourne University and TUSZ have the largest total dataset size and this contributed to having more preictal segments or seizure events. While Kaggle UPenn has the highest total recorded segments, it contains only 28 seizure events due to the length of each segment, which is just 1 second. Similarly, while the Siena Scalp EEG dataset contains fewer recorded segments than the Kaggle UPenn dataset, it has 47 ictal segments due to the longer length of each segment.

### 6.3 Sampling frequency

Lastly, like the other temporal properties, the sampling frequency also varies across the datasets. Different sampling frequencies also exist within the same dataset in Kaggle UPenn, AES seizure, and TUSZ dataset with the first two having a range of between 400 and 500 Hz while the latter having a minimum of 250 Hz for each of the segments. The remaining datasets have constant sampling frequency within the dataset, with the highest sampling frequency of 512 Hz in Siena Scalp EEG and the lowest in the University of Bonn at 173.6 Hz. Sampling frequency of the datasets can also be found in Table 8.

## 7 DISCUSSION

The findings of the datasets summarized above enable both clinicians and ML researchers to make an informed decision about the algorithm and model based on the task. This section explains how the characteristics of the current datasets might impact the algorithms and models proposed by clinicians and ML researchers.

First, it is important to note that the dataset from the Melbourne University competition and Neurovista ictal datasets are obtained from the same recording based on the long‐term continuous intracranial recordings from the first‐in‐man long‐term seizure prediction trial,^5^ with both datasets available at https://www.epilepsyecosystem.org/. The dataset from Kaggle Melbourne University's competition includes data from the 3 patients who performed worst in the first‐in‐man seizure prediction trial study. Hence, studies intending to use both of the datasets need to take note to prevent data leakage producing inaccurate results.

Some of the datasets such as the University of Bonn and Hauz Khas or private datasets only include specific channels that are less reflective of real‐world EEG data. Studies that cite datasets with specifically preselected channels such as channels within the epileptogenic zone achieve higher prediction and detection performance.^27^ However, this type of dataset is less reflective of real‐world EEG recordings involving multiple channels across different parts of the brain. Hence, this type of dataset reduces the feasibility of a model to be implemented in seizure detection or prediction devices even if the performance of the model is deemed great.

The variation in terms of the number of channels used might prevent a model trained on a specific dataset to be tested on other datasets. This is because the number of channels in a dataset directly affects the total number of features used to train a traditional ML model where the total number of features is generally calculated with the formula:

$$\text{Number of channels}\;\left(c\right)\;x\;\text{number of bands}\;\left(b\right)\;x\;\text{features type}\;\left(f\right)$$

where *c* depends on the dataset itself, whereas *b* and *f* are dependent on the authors of a study. So, the dataset used for training and testing needs to include the same number of channels and similar electrode placement methods. One way to overcome this issue is to select datasets that have a similar electrode placement and a number of channels that follow the International 10–20 EEG placement. However, scientists and clinicians need to take note that datasets that follow a similar electrode placement method might still have a different number of channels with additional electrodes or channels to increase EEG resolutions. Some datasets, such as those of TUSZ and Siena Scalp EEG have additional channels compared with the Helsinki University Hospital EEG dataset, while still following the international 10–20 EEG placement due to them including channels from the Nomenclature system, producing more detailed EEG recordings.^28^ Further, while scalp datasets tend to follow the International 10–20 system placement, the naming of each channel in each of the datasets is different, and there is a mismatch in the number of channels in the original studies, databases, and datasets, which can be observed in references to the Siena Scalp EEG and CHB‐MIT datasets. Hence, future seizure prediction or detection studies using the dataset should follow the instruction provided by the original studies or documentations when choosing the channels needed for seizure prediction or detection tasks.

Scalp EEG recording is easy to obtain in clinical settings and is relatively cheap compared with intracranial EEG. Intracranial EEG (iEEG) signals are recorded by electrodes implanted on the surface of the cerebral cortex. As discussed earlier, iEEG signals have a higher signal‐to‐noise ratio compared with surface EEG as they are less affected by artifacts and environmental factors.^1^, ^29^ Further, iEEG recordings are able to capture more epileptiform discharges compared with scalp recordings due to high signal attenuation by the skull from the source to the scalp.^30^ Studies with algorithms that are intended to be used in ICUs or when time is a concern, should use scalp or surface EEG datasets when training their model, since it is relatively fast and easy to be implanted and there is a low risk of injuries. Studies with algorithms that are intended to be implemented in a long‐term seizure warning or detection device for epilepsy patients for use outside of a hospital environment should use an intracranial dataset since it has a higher signal‐to‐noise ratio and contains fewer artifacts caused by movements and signal drops.

Since characteristics of preictal or seizure biomarkers are different and the EEG characteristics of seizures show variation across all patient groups and seizure types,^5^, ^18^, ^31^ using datasets with a low number of subjects or small volume of data, where the EEG recordings of all patients are grouped together to detect seizure patterns or characteristics outside the patient's group reduces the chance of a generalized model. The generalized model only learns features and biomarkers present in the current group of patients but might fail to highlight or capture important unique characteristics of certain patients outside the group. Increasing the variation of the characteristics of seizure biomarkers by choosing a dataset with a larger number of subjects like TUSZ with a higher number of seizure events might allow the model to better recognize seizure patterns when tested with other datasets. However, using larger datasets or multiple datasets for a certain study introduces potential complexity if there are differences in the number of channels or structural properties.

Looking at the temporal properties of the dataset, seizures in patients tend to show a pattern in relation to the time. It has been shown that seizure likelihood also increases if a seizure occurred recently and in certain groups of patients, cyclic trends such as the timing of the day can also be found in periods leading to seizures.^32^, ^33^, ^34^, ^35^ Datasets that contain at least 24 hours of data for some patients such as CHB‐MIT allow a seizure detection and prediction model, which considers the time component in their algorithm. This can be seen in studies using where the model with a circadian component using long‐term continuous Neurovista dataset performs better than those without it.^4^, ^36^ However, the long‐term continuous dataset is not publicly available to use, but the partial dataset is represented in the Kaggle Melbourne University dataset and Neurovista ictal dataset. The use of datasets with short recording or lacking temporal information is hence limited to algorithms that do not use time components.

Most of the datasets discussed are imbalanced, i.e., they have a significantly higher proportion of interictal events compared with ictal or preictal segments. This might affect the performance of several popular models susceptible to class imbalance, such as neural networks and Support Vector Machines (SVM), producing results that are highly unreliable and poor performance in testing environments if the minority class is not fully represented in the data.^37^, ^38^ While balancing datasets through resampling techniques might help to solve the problem of class imbalance in ML where several techniques have been used in seizure detection and prediction,^16^, ^39^, ^40^ scientists and engineers need to be aware that a balanced dataset does not represent the true nature of EEG recording where the chance of seizures happening during recording is relatively rare in epilepsy patients.^41^ The choice of an ML model and algorithm will also largely depend on the dataset. Boosting or bagging techniques that consist of an ensemble of weak classifiers or cost‐sensitive models that consider the misclassification cost of each class, will often perform better than other models on datasets that are imbalanced.^42^, ^43^ Additionally, some smaller datasets like the Bonn University and Hauz Khas datasets are not suited to be trained on deep learning models without preprocessing or data augmentation since deep learning models require a high volume of data to tune the parameters in the learning model.^44^, ^45^

While patients' medical information might be difficult to interpret in an ML model due to format constraints, clinical information such as type of seizures, EEG characteristics, and the area of seizure onset might help a model to better recognize seizures.^46^, ^47^ ML models with natural language processing components might be able to interpret medical records of each patient to better tune the model to detect or predict patient‐specific seizures. Using EMG‐based seizure detection devices attached on specific body parts such as the arms, studies have demonstrated the ability to detect generalized tonic–clonic seizures (GTCS) to detect seizures with sensitivities of more than 90%.^48^, ^49^ Further, studies have shown that there are significant changes in the heart rate such as tachycardia and increased heart rate variability.^50^, ^51^ Using datasets that include additional biosignals such as ECG or EMG may help improve the performance of seizure detection and prediction algorithms.

Lastly, there are huge variations across several characteristics that prevent studies from truly testing their trained algorithm on another dataset without re‐tuning the algorithm. Different sampling frequencies and the length of each epoch in a segment might not truly reflect the model's performance in out‐of‐sample tests or when several datasets were used to compare the model's performance, even if it underwent additional preprocessing steps. Moreover, the different temporal properties of the datasets (i.e., long‐term continuous dataset, short‐term continuous dataset, and noncontinuous dataset) will require different preprocessing methods, and this will result in a discrepancy in the performance if two datasets with different characteristics or temporal properties are tested.

## 8 LIMITATIONS

While we managed to include all characteristics of an EEG dataset, the narrative review faced a few limitations. The total seizure time for patients in each of the datasets was not calculated due to time constraints and the need to access and process each dataset. While studies such as TUSZ included the total seizure duration, most datasets did not. Hence, clinicians and scientists interested in total seizure duration would have to extract the information from the seizure segments. In practice, prediction and detection algorithms will still need to handle variable‐length seizures. While some datasets included information on the type of seizures, we did not include the type of seizures that are present in each of the datasets. Information on the type of seizures is included in each patient's description and stored in a nonstandard structure for each dataset, which is time‐consuming to manually extract. However, researchers who are interested in developing algorithms that are specific to seizure type can use Table 6 as a guide to select datasets with information on seizure type.

Another limitation is the discrepancy in the unit of measurement provided for some of the characteristics across the datasets, such as the use of a number of “seizure segments” or “seizure events” depending on the datasets. This might cause confusion when comparing the datasets since some might consider seizure segments and seizure events as the same definition. Some of the scalp datasets include other channels that we were not able to deduce due to unrecognizable names and a lack of information in the documentation and studies. It should also be noted that dataset like Siena Scalp EEG contains other signals such as oxygen saturation (SPO2) containing values of 0. However, we do not consider this a major limitation, since EEG signals are the main focus and the scalp datasets normally follow the international 10–20 system or the Nomenclature 10–10 system.

Using the citation count to select studies may lead to bias, as studies that report positive results tend to be cited more often.^52^ In our study, the effect of this bias is minimal, as we were only interested in how many of the studies use the data rather than the results themselves. However, there could potentially be a bias for small/poorly designed datasets (i.e., with low numbers of seizures or data durations per patient) to be cited more often, as the results obtained on these datasets will appear more impressive (e.g., near 100% sensitivity as a result of overfitting to a small dataset).

## 9 CONCLUSION AND FUTURE WORK

In this narrative review, we present the key publicly available datasets and the characteristics of each of those datasets. The findings and key issues presented for each of the datasets in the discussion section allow clinicians and engineers to consider and make an informed decision on whether it is a seizure prediction or detection task and the selection of appropriate ML algorithm. Instead of looking for a novel ML algorithm, considering the issues and the characteristics of the EEG dataset may allow us to move closer to detecting and predicting seizures for the epilepsy community and move forward to clinical trials using already proposed algorithms developed by scientists and clinicians.

Future work could include performing a more detailed survey of how studies make use of the data. Specifically, what properties of the data do studies depend on, what other assumptions do they make about datasets (e.g., assume a balanced dataset), and what techniques they used to overcome some of the issues of the dataset. These could also extend to formalizing the analysis presented into a characterization model that allows EEG datasets to be annotated with additional information across multiple dimensions, covering both the data and ML techniques for an easier assessment of suitability.

## CONFLICT OF INTEREST STATEMENT

S. Sivathamboo is supported by a Bridging Postdoctoral Fellowship from Monash University (BPF20‐3253672466) and the Victorian Medical Research Acceleration Fund. She reports salary support from Kaoskey and Optalert for clinical trial‐related activities; she receives no personal income for these activities. P. Perucca is supported by the National Health and Medical Research Council (APP1163708), the Epilepsy Foundation, The University of Melbourne, Monash University, Brain Australia, and the Weary Dunlop Medical Research Foundation. He has received speaker honoraria or consultancy fees to his institution from Chiesi, Eisai, LivaNova, Novartis, Sun Pharma, Supernus, and UCB Pharma, outside the submitted work. He is an Associate Editor for Epilepsia Open.P. Kwan is supported by a Medical Research Future Fund Practitioner Fellowship (MRF1136427) and the Victorian Medical Research Acceleration Fund. He reports grants and personal fees from Eisai, UCB Pharma, and LivaNova; reports grant from Zynerba, Biscayne, and GW Pharmaceuticals. L. Kuhlmann is supported by the National Health and Medical Research Council (GNT1183119; GNT1160815), the Epilepsy Foundation of America, and the Australian Research Council (DP210100045; DP200102600). T. O'Brien is supported by a Program Grant from the National Health and Medical Research Council of Australia (APP1091593) and the Victorian Medical Research Acceleration Fund. He reports grants and personal fees from Eisai, UCB Pharma, and Zynerba. None of the other authors has any conflict of interest to disclose. We confirm that we have read the Journal's position on issues involved in ethical publication and affirm that this report is consistent with those guidelines.

## APPENDIX A

**TABLE A1:** Usage frequency of datasets by studies.

Dataset	Count
University of Bonn	58
CHB‐MIT Scalp EEG	31
Freiburg	19
Neurovista	4
The European Epilepsy Database	3
Kaggle UPenn and Mayo Clinic's Seizure Detection Challenge	3
Neurology and Sleep Centre Hauz Khas	3
Xi'an Jiaotong University	2
Cork University Maternity Hospital	2
Kaggle American Epilepsy Society Seizure Prediction Challenge	2
Sir Ganga Ram	2
Royal Brisbane and Women's Hospital	2
Nanyang Technological University	1
The Mount Sinai Epilepsy Center	1
Osaka University Hospital	1
Karunya University	1
Peking University People's Hospital	1
Spectrum Health, Grand Rapids	1
University College London Hospital	1
Seoul National University Hospital	1
Hospital Regional Universitario Carlos Haya	1
